# Remission of social behavior impairment by oral administration of a precursor of NAD in CD157, but not in CD38, knockout mice

**DOI:** 10.3389/fimmu.2023.1166609

**Published:** 2023-05-04

**Authors:** Maria Gerasimenko, Haruhiro Higashida

**Affiliations:** Department of Basic Research on Social Recognition and Memory, Research Center for Child Mental Development, Kanazawa University, Kanazawa, Japan

**Keywords:** NAD, nicotinamide riboside, CD157, CD38, social behavior, neuromodulator

## Abstract

Nicotinamide adenine dinucleotide (NAD) is a substrate of adenosine diphosphate (ADP)-ribosyl cyclase and is catalyzed to cyclic ADP-ribose (cADPR) by CD38 and/or CD157. cADPR, a Ca^2+^ mobilizing second messenger, is critical in releasing oxytocin from the hypothalamus into the brain. Although NAD precursors effectively play a role in neurodegenerative disorders, muscular dystrophy, and senescence, the beneficial effects of elevating NAD by NAD precursor supplementation on brain function, especially social interaction, and whether CD38 is required in this response, has not been intensely studied. Here, we report that oral gavage administration of nicotinamide riboside, a perspective NAD precursor with high bioavailability, for 12 days did not show any suppressive or increasing effects on sociability (mouse’s interest in social targets compared to non-social targets) in both CD157KO and CD38KO male mice models in a three-chamber test. CD157KO and CD38KO mice displayed no social preference (that is, more interest towards a novel mouse than a familiar one) behavior. This defect was rescued after oral gavage administration of nicotinamide riboside for 12 days in CD157KO mice, but not in CD38KO mice. Social memory was not observed in CD157KO and CD38KO mice; subsequently, nicotinamide riboside administration had no effect on social memory. Together with the results that nicotinamide riboside had essentially no or little effect on body weight during treatment in CD157KO mice, nicotinamide riboside is less harmful and has beneficial effect on defects in recovery from social behavioral, for which CD38 is required in mice.

## 1 Introduction

Prosocial behavior is important for humans and in the human diverse society (1–3), and humans naturally possess social cognition and memory ability at different levels (4, 5). Social cognition is a behavior that covers aspects of information about one’s self and others within their social groups (4, 6). Alternatively, social recognition is an ability to discriminate familiar and unfamiliar social objects and to interact between them, which is processed as social memory. Therefore, such abilities are required for living in a society to learn members’ identities, to maintain groups between friendly individuals or at the working places, and for interpersonal communication. In our society, we need to acquire social skills to make social decisions (2, 3).

In experimental neurosciences, social recognition is usually defined as an interest towards novel social objects (social motivation), and social memory is defined as a decrease in investigative behaviors toward re-exposed (and thus have become) familiar conspecifics (7, 8). Among the various neurotransmitters involved in social recognition, oxytocin is reported to be involved in social interaction, social recognition, and memory in the social brain (9). Disruption of the oxytocin system leads to impaired social recognition and mutual interactions in humans with psychiatric disorders, such as autism spectrum disorders (ASDs) or schizophrenia (10–12). Defects in the oxytocin system is involved in anxiety-, depression-, avoidance-, and hyperactivity-like behaviors, which are useful psychiatric disorder models (13, 14). For the past 20 years, studies have shown that CD38 and CD157 are critical molecules in prosocial behavior (9, 14–28), as described below.

Oxytocin is synthesized in neurons in the paraventricular nucleus and supraoptic nucleus of the hypothalamus, and secreted somato-dendritically from oxytocin-producing neurons into the brain. It plays the role of a neuromodulator (24, 29, 30). CD38 is a cell-surface antigen with adenosine diphosphate (ADP)-ribosyl cyclase activity, which catalyzes cyclic ADP-ribose (cADPR) from nicotinamide adenine dinucleotide (NAD) (31, 32). cADPR functions as a second messenger to trigger Ca^2+^ mobilization from endoplasmic Ca^2+^ pools (31, 33). In the hypothalamus, cADPR elevates intracellular free Ca^2+^ concentrations and subsequently releases oxytocin from oxytocinergic neurons (24). The linkage between this signaling cascade and social behavior was, for the first time, shown in *Cd38* knockout (CD38KO) mice, in which social memory and recognition or parental nurturing behavior were disrupted mainly owing to reduced oxytocin secretion (27). The importance of CD38 and oxytocin in social memory was further confirmed by local re-expression of human CD38 in the hypothalamus by *CD38*-containing lentivirus infection or simple subcutaneous supply of oxytocin in CD38 KO mice, in which social behavioral impairment was rescued (28).

CD157 (originally found as BST-1) (34) is a sister molecule of CD38 and is expressed in neuroprogenitor or neurolineage cells in the subventricular zone of the fetal brain (21). The functional role of CD157 on oxytocin’s reaction is nearly identical to that of CD38. CD157 produces cADPR at one third of the CD38’s catalyzing level but with almost no nicotinic acid adenine dinucleotide phosphate producing ability, differing from CD38. Thus, various functions of CD157 seem to be mediated by not only cADPR with respect to Ca^2+^ homeostasis, but also by migrating powers with homophilic binding on the cell surface or adhesive properties associated with integrin (35). Though the phenotypes of *Cd157* knockout (CD157KO) and CD38KO mice in social behavior are largely similar, CD157 invokes multiple circuits to control anxiety- and depression-like behaviors (14, 20, 21).

Nicotinamide riboside, a NAD precursor, is converted to nicotinamide mononucleotide through nicotinamide riboside kinase 1. Subsequently, nicotinamide mononucleotide is converted to NAD by the action of nicotinamide mononucleotide adenylyl transferase. Nicotinamide riboside kinase 1 and nicotinamide riboside kinase 2 genes are utilized in a *de novo* fashion (36–39). Evidence shows that nicotinamide riboside supplementation in humans increases intracellular NAD concentrations and subsequently improves NAD-dependent activities in the cell by increasing silent mating-type information through nicotinamide riboside kinase 2-dependent gene silencing, and longevity *via* nicotinamide riboside kinase 1-dependent NAD synthesis (39, 40). Thus, it is possible that the exogenous application of nicotinamide riboside can promote the biosynthesis of NAD *via* nicotinamide mononucleotide. Subsequently, NAD is degraded to cADPR and nicotinamide by poly(ADP-ribose) polymerases (PARPS) and CD38, respectively. cADPR helps produce the beneficial effects of NAD (41). The most fundamental use of NAD precursor molecules, nicotinic acid and nicotinamide mononucleotide, is the prevention of pellagra. Similar to nicotinic acid and nicotinamide mononucleotide, nicotinamide riboside is a natural product found in milk (38), which is incorporated into the intracellular NAD pool (42–44), and thus can be used as a general supplement, potentially for people who have adverse reactions to nicotinic acid or nicotinamide mononucleotide. However, more significantly, the specific utilization of nicotinamide riboside by neurons may provide qualitative advantages over niacin in promoting function in the central and peripheral nervous systems. Nicotinamide riboside has been already used as a supplement or therapeutic agent to elevate or maintain cellular NAD contents because of increase in CD38 in aged subjects (45, 46).

Nicotinamide riboside is beneficial for treating social impairments in young and aged people, some of which are based on impairments of oxytocin and/or oxytocin release (17); however, this finding is less reported. To assess this question, we previously investigated the role of orally applied nicotinamide riboside (gavage route) for 12 days on impaired social behaviors in CD157KO mice. We re-examined the effects of gavage administration of nicotinamide riboside in CD157KO mice by using a single protocol (17). Furthermore, we studied the effect of nicotinamide riboside in CD38KO mice. These comparative results clarified the functional roles of CD157 and CD38 as neuromodulators, rather than immune factors in diseases including cancer (36, 47).

## 2 Methods

### 2.1 Animals

CD157KO (*Cd157^-/-^*/*Cd38^+/+^* of the C57BL/6 genetic background) and CD38KO (*Cd157^+/+^*/*Cd38^-/-^* of the ICR genetic background) mice were created as described previously (28, 34, 48, 49), and were kindly provided by Ishihara and Okamoto, respectively. The mice were maintained by crossbreeding homozygous mutant mice. Slc : ICR (CD-1) outbred male mice (8 weeks old, 25–30 g body weight) and C57BL6/N (8 weeks old, 23–27 g body weight) mice were obtained from Japan SLC Inc. (Hamamatsu, Japan) through a local distributor (Sankyo Laboratory Service Corporation, Toyama, Japan) and used as controls for CD38KO and CD157KO mice, respectively. Half of the offspring of wild-type mice and all KO mice were bred in our laboratory colony, weaned at 21–32 days of age, and housed in same-sex groups of five sibling pairs in one cage in the animal center under standard conditions (24°C; 12/12-h light/dark cycle, with lights on at 8:45 a.m.) with food and water *ad libitum*. After pretest and during the test each mouse was housed in individual cage.

All animal experiments were carried out in accordance with the Fundamental Guidelines for Proper Conduct of Animal Experiment and Related Activities in Academic Research Institutions under the jurisdiction of the Ministry of Education, Culture, Sports, Science and Technology of Japan and approved by the Committee on Animal Experimentation of Kanazawa University Graduate School of Medical Sciences.

### 2.2 Animal treatment

Nicotinamide riboside was supplied by Brenner from ChromaDex, Irvine, CA, USA. Male mice were gavage administered with nicotinamide riboside in a dose of 13 mg in a 50 μl solution, or the equivalent volume of physiological saline (PBS) as placebo control (17). Mice were treated daily at around 3-5 p.m. for 12 days.

### 2.3 Social behavior test in three-chamber boxes

The sociability, social preference, and social memory were tested using a three-chamber box to assess whether subject mice tended to spend time with mice in different chambers, as described previously (50) (**Supplementary Figure 1**).

#### 2.3.1 Habituation

Test mice were first habituated for 5 min in an empty three-chamber box.

#### 2.3.2 Sociability

Sociability was examined in a 5-min interval by the duration for which an experimental naive male mouse stayed in either a left chamber with a non-social target (usually a 50-ml conical plastic tube) or in the right chamber with a male mouse in a small metal mesh cage. Usually, the mouse stays longer with the target mouse due to its prosocial nature.

#### 2.3.3 Social preference

At the end of the 5-min sociability test, these subject and target mice were immediately tested in a third 5-min session to quantitate the preference to spend time with a new unfamiliar mouse (Stranger 2; an experiment naïve male) placed in the wire cage (in the right chamber), replacing the plastic tube in the previous 5-min session. The test mouse had a choice to stay with either the first (already investigated, now familiar mouse; Stranger 1) or the novel unfamiliar mouse (Stranger 2). The mice usually interact more with the unfamiliar mouse.

#### 2.3.4 Social memory

At the end of the 5-min social preference test, the mouse (Stranger 1) in the previous stage was utilized in another (fourth) 5-min session to quantitate social memory after 30 min. It was examined if the test subject spent more time with a second new stranger (Stranger 3; an experiment naïve male) than Stranger 1 to test for short-term social memory after posing the 30-min separation. Stranger 3 was placed in the wire cage (in the right chamber) that had been occupied by Stranger 2 during the previous 5-min session, and after 30 min separation. The test mouse had a choice between the first, already-investigated, now-most familiar mouse (Stranger 1) and the novel unfamiliar mouse (Stranger 3).

The trial was recorded for 5 min using the ANY-maze video system, as described previously (14, 17). Latency to enter (defined by all four paws entering), time spent, entries, and distance travelled in the light chamber were recorded. Experiments were repeated thrice on one day after treatment for 12 days.

### 2.4 Statistical analysis

The data are expressed as the means ± standard error of mean. The comparisons were evaluated using Student’s *t*-test and One-way or Two-way ANOVA, followed by *post-hoc* Bonferroni test. In all analyses, *P* < 0.05 indicated statistical significance.

## 3 Results

### 3.1 On CD157 knockout mice

**Figure 1:**
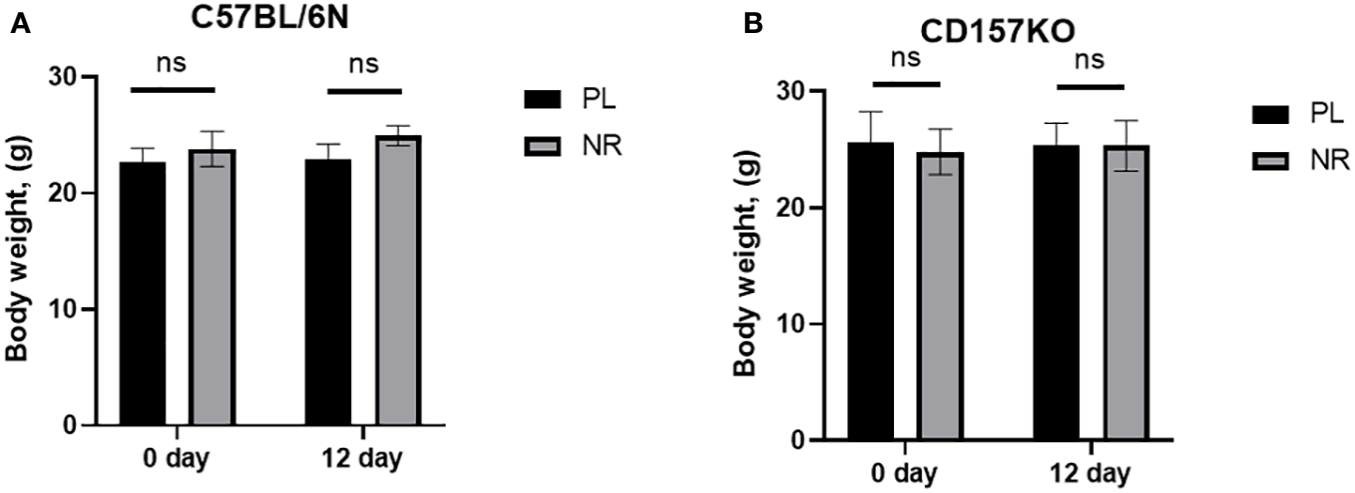
Nicotinamide riboside (NR) treatment has no effect on mouse body weight. **(A)** Body weight of C57BL6 male mice, before (0 day) and after NR (13 mg/mouse) or placebo (PL) treatment for 12 days (PL: n = 6, unpaired *t*-test, *t*(10) = 0.3571, *P* = 0.7285; NR: n = 6, *t*(10) = 1.598, *P* = 0.1412). **(B)** Body weight of CD157 KO mice before (0 day) and after NR or placebo (PL) treatment for 12 days (PL: n = 6, unpaired *t*-test, *t*(10) = 0.2399, *P* = 0.8152; NR: n = 6, unpaired *t*-test, *t*(10) = 0.4341, *P* = 0.6734).

Adult male C57BL6/N wild-type and CD157KO mice treated with saline or 13 mg nicotinamide riboside (approximately 350-400 mg/kg of body weight) for 12 days did not demonstrate any apparent changes in coordinated movement dysfunction (data not shown). The body weight gain during gavage had no or little difference between treatments with saline and nicotinamide riboside (**Figures 1A, B** **)**.

### 3.2 Social interaction in the three-chamber box test in CD157KO mice

**Figure 2:**
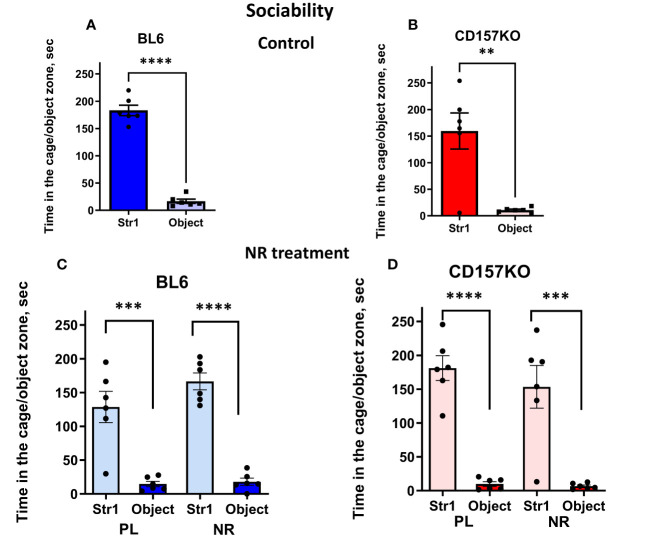
Nicotinamide riboside **(**NR) treatment has no apparent effect on sociability in C57BL6 (BL6) and CD157KO male mice in the three-chamber test. Duration spent in the chamber with Stranger 1 mouse in a cage (Str1) or a non-social target (object) by wild-type (BL6; **A**) or CD157KO **(B)** mice. **(C, D)** Duration spent in the chamber with Stranger 1 mouse in a cage (Str1) or a non-social target (object) by wild-type (BL6; **C**) or CD157KO **(D)** mice, which were treated with gavage administration of saline (PL) or nicotinamide riboside (NR) for 12 days and then examined. Two-tailed Student’s *t*-test, **P < 0.01, ***P < 0.001, ****P < 0.0001.

We performed three types of social interaction tests with novel social targets each time (that is, sociability, social preference, and social memory). A target adult male mouse was placed in the left chamber (Stranger 1) and a non-social target in the right chamber, under which condition we could test sociability (mouse’s general interest towards social targets). Wild-type and CD157KO mice interacted with the social target mouse (Stranger 1) significantly longer than with non-social targets (two-tailed Student’s *t*-test, *P* < 0.0001 for both; **Figures 2A, B** **)**. The result indicated that the typical phenotype of the social mouse, called sociability, was not disrupted even in CD157KO mice. This sociability phenotype in both genotypes was not much affected by gavage treatment of saline (placebo) or nicotinamide riboside (13 mg/mouse) daily for 12 days (**Figures 2C, D**; two-tailed Student’s *t*-test, *P* < 0.001), similar to previously reported results (17).

**Figure 3:**
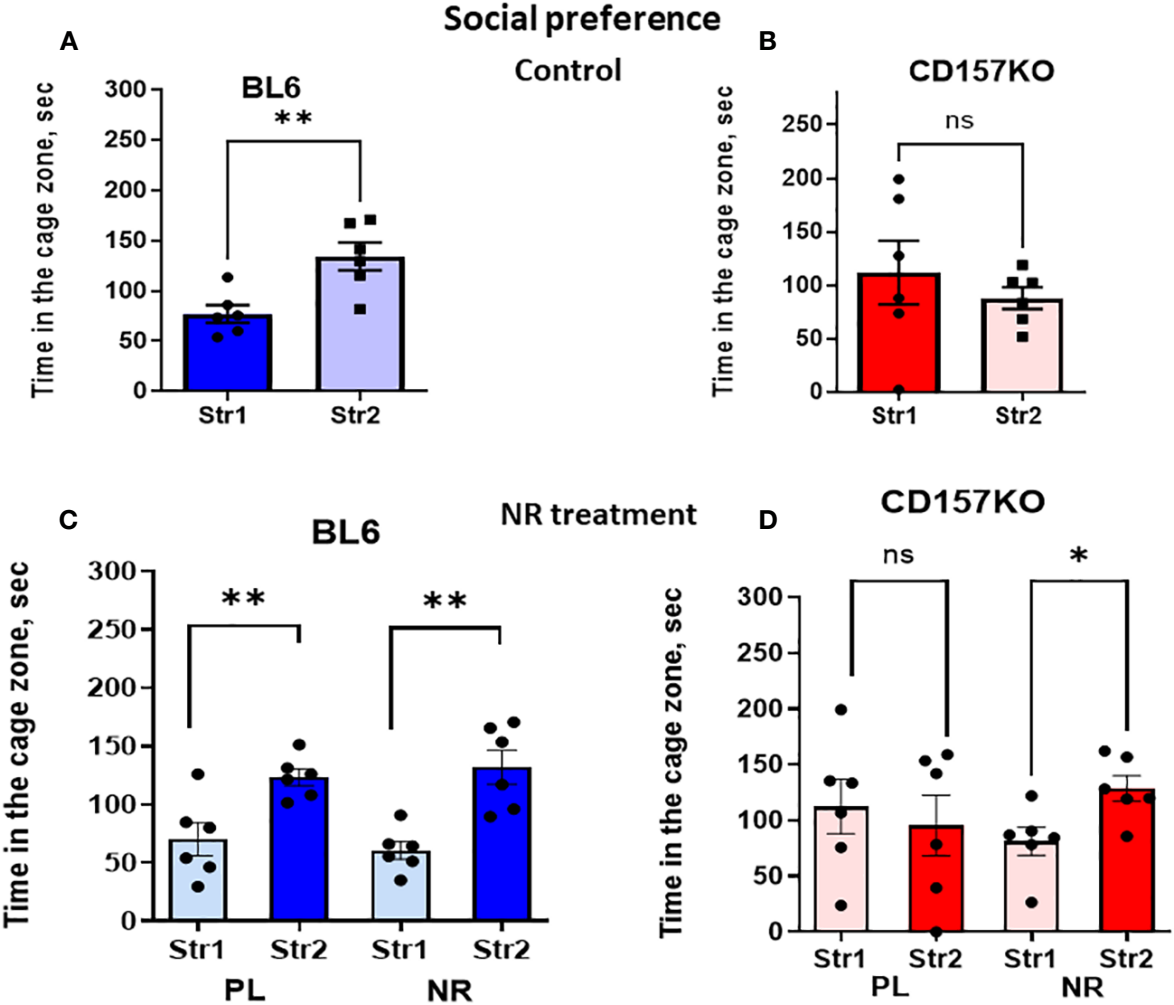
Nicotinamide riboside (NR) treatment corrects social preference deficit in CD157KO mice. Duration spent in the chamber with the familiar mouse in a cage (Str1) or a novel mouse (Str2) by wild-type (BL6; **A, C**) or CD157KO **(B, D)** mice. Mice were treated with gavage administration of saline (PL) or nicotinamide riboside (NR) for 12 days **(C, D)** or were without any treatment **(A, B)**. Two-tailed Student’s *t*-test, *P < 0.05, **P < 0.01. ns, not significant.

The non-social target was replaced with the second new target male mouse in the right chamber (Stranger 2) to examine social novelty (social preference between familiar (Stranger 1) and new unfamiliar (Stranger 2) mice; **Supplementary Figure 1**; **Figure 3**). **Figure 3A** shows the time spent in the zone with Stranger 2 is significantly longer for wild-type mice (two-tailed Student’s *t*-test, *P* < 0.0001). Contrastingly, in CD157KO mice, the time spent in the two zones are similar (**Figure 3B**). The result indicated that, unlike in the wild-type mice, social preference to new social targets was completely lost in CD157KO mice.

No social preference phenotype in CD157KO mice was changed after gavage treatment with saline for 12 days (**Figure 3D**). In sharp contrast, CD157KO mice treated with gavage nicotinamide riboside (13 mg daily for 12 days) interacted much more frequently with Stranger 2 compared with stranger 1 (**Figure 3D**); the time spent in the area with Stranger 2 was significantly longer than that in the zone with Stranger 1 (two-tailed Student’s *t*-test, *P* < 0.01). Wild type mice treated with nicotinamide riboside or saline displayed significant social preference (two-tailed Student’s *t*-test, *P* < 0.01; **Figure 3C**).

**Figure 4:**
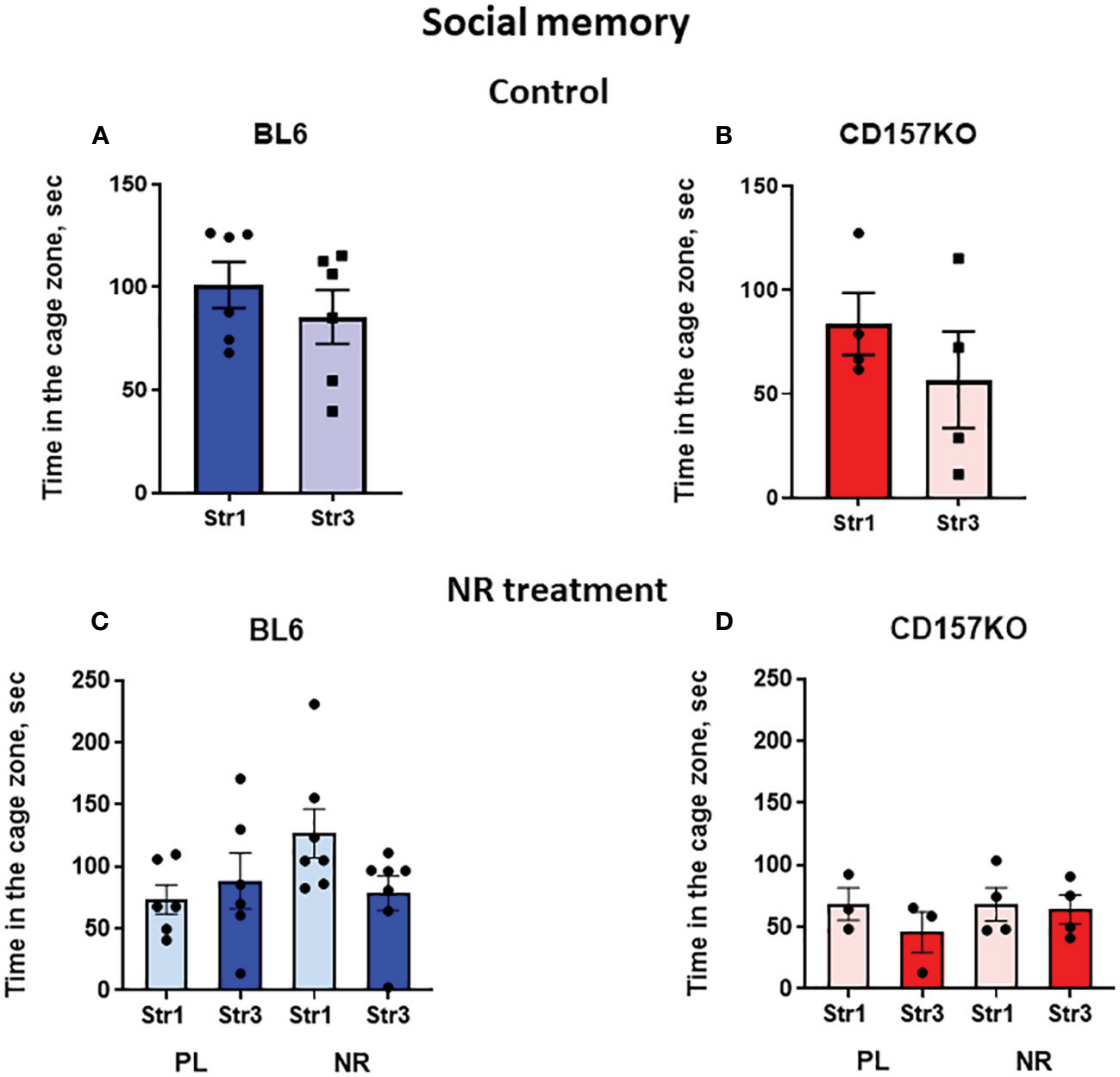
Wild-type C57BL6 (BL6) and C157KO mice show absence of social memory and no or little effect of nicotinamide riboside (NR) treatment. Duration spent in the chamber with the familiar mouse in a cage (Str1) or a novel mouse (Str3) by wild-type (BL6; **A, C**) or CD157KO **(B, D)** mice. Mice were treated with gavage administration of saline (PL) or NR for 12 days **(C, D)** or were without any treatment **(A, B)**.

In the social memory stage, the Stranger 2 mouse was replaced with a novel social stimulus (Stranger 3) (**Supplementary Figure 1** **).** Next behavior tests were performed after 30 min, which allowed to measure short-term memory in mice. Both genotypes of wild-type and CD157KO mice lacked significant social memory, without any preference to stranger 3 compared to Stranger 1 (**Figures 4A, B**); nicotinamide riboside did not affect this preference (**Figures 4C, D** **)**.

### 3.3 On CD38 knockout mice

**Figure 5:**
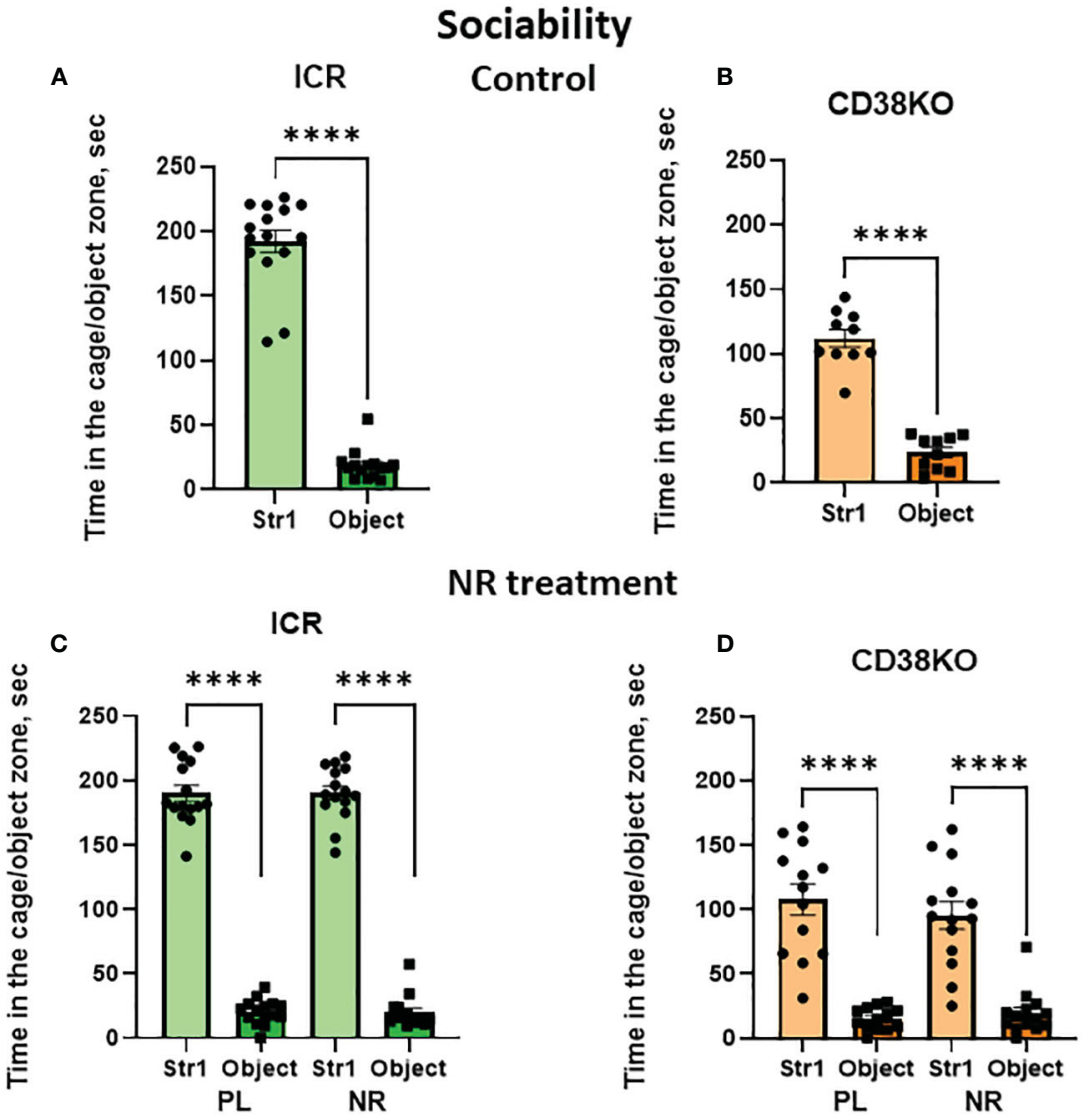
Nicotinamide riboside (NR) treatment has no apparent effect on sociability in wild-type (ICR) and CD38KO male mice in the three-chamber test. Duration spent in the chamber with a novel mouse in a cage (Str1) or a non-social target (object) by ICR **(A, C)** or CD38KO **(B, D)** mice. Mice were treated with gavage administration of saline (PL) or NR for 12 days **(C, D)** or were without any treatment **(A, B)**, and then were examined for sociability behavior. Two-tailed Student’s *t*-test, ****P < 0.0001.

Finally, we examined the effects of nicotinamide riboside in CD38KO mice to assess whether CD38 was necessary for the recovery of social behavior defects. As done with the CD157KO mice, social behavior was examined in the three-chamber box test. CD38KO mice displayed the sociability phenotype, similar to the wild-type ICR mice (**Figures 5A, B** **)**. This sociability in CD38KO mice was unaffected by gavage administration of saline (Student’s *t*-test *P* < 0.0001) or treatment with nicotinamide riboside (Student’s *t*-test *P* < 0.0001; **Figures 5C, D** **)**.

**Figure 6:**
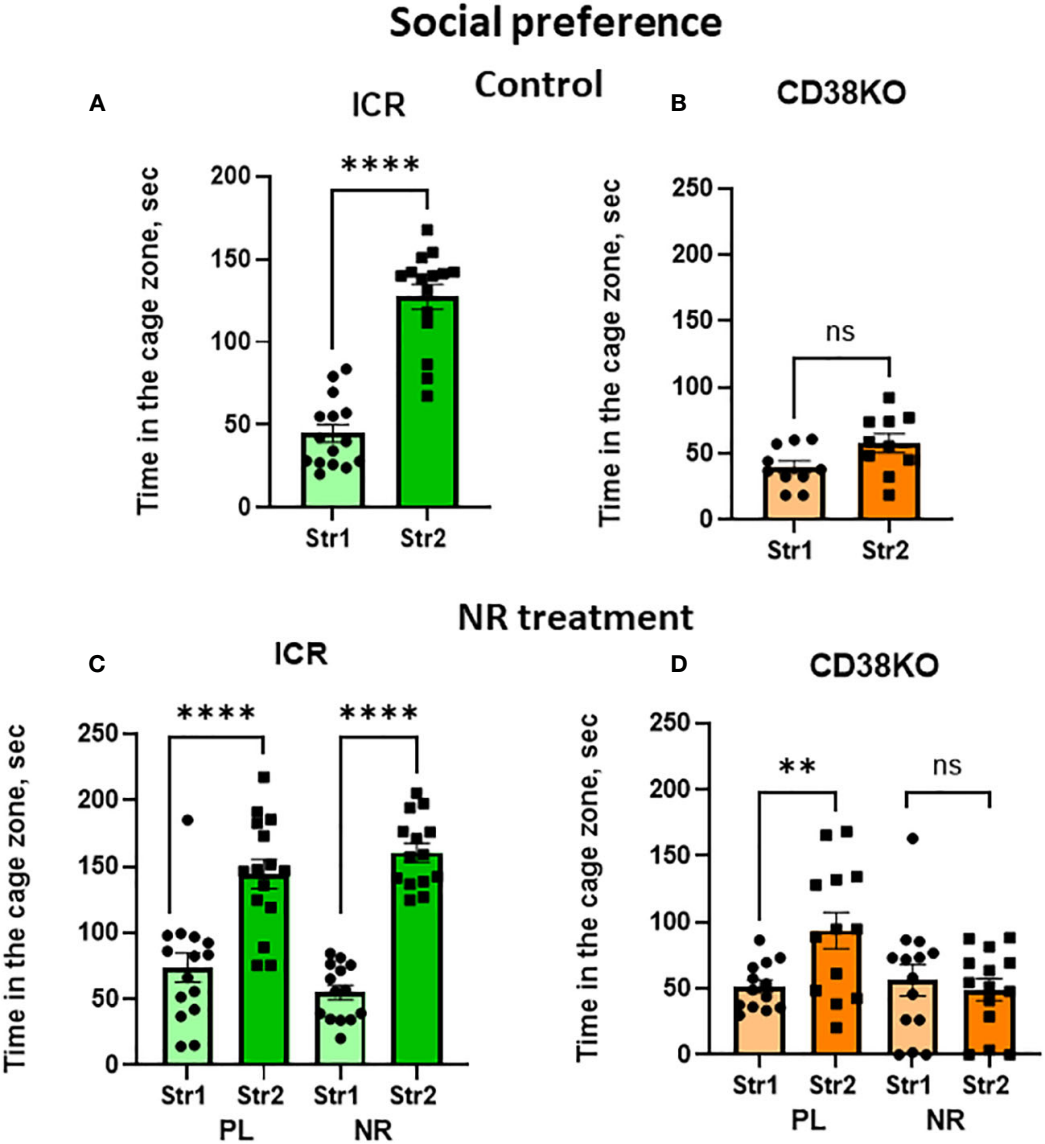
Effects of nicotinamide riboside (NR) or saline (PL) treatment on social preference behavior in wild-type (ICR) or CD38KO mice. Duration spent in the chamber with the familiar mouse in a cage (Str1) or a novel mouse (Str2) by wild-type **(A, C)** or CD38KO **(B, D)** male mice. Mice were treated with gavage administration of saline (PL) or NR for 12 days **(C, D)** or were without any treatment **(A, B)**. Two-tailed Student’s *t*-test, **P < 0.01, ****P < 0.0001. ns, not significant.

Unlike the wild-type mice (**Figure 6A**; Student’s *t*-test *P* < 0.0001), CD38KO displayed no or little social preference (**Figure 6B**; Student’s *t*-test *P* = 0.055). Such social preference was unaffectedly observed in wild-type mice under treatment with saline as well as with nicotinamide riboside (Student’s *t*-test *P* < 0.0001; **Figure 6C**). CD38KO mice treated with gavage administration of nicotinamide riboside (13 mg/day) for 12 days showed a complete lack of social preference (two-tailed Student’s *t*-test, *P* > 0.05; **Figure 6D**). Unexpectedly, significance in social preference was observed in CD38KO mice treated with saline gavage (**Figure 6D**; two-tailed Student’s *t*-test, *P* < 0.01). The reasons for why such unexpected result was obtained are not clear, but one reason may reside on the handling effect of mice during gavage, because handling animals sometimes causes undesirable results is well reported (51). Note that, although some activities in the central zone were lower in CD38KO mice, probably because of moving around other places due to hyperactive features (24, 28), CD38KO mice displayed the sociability.

**Figure 7:**
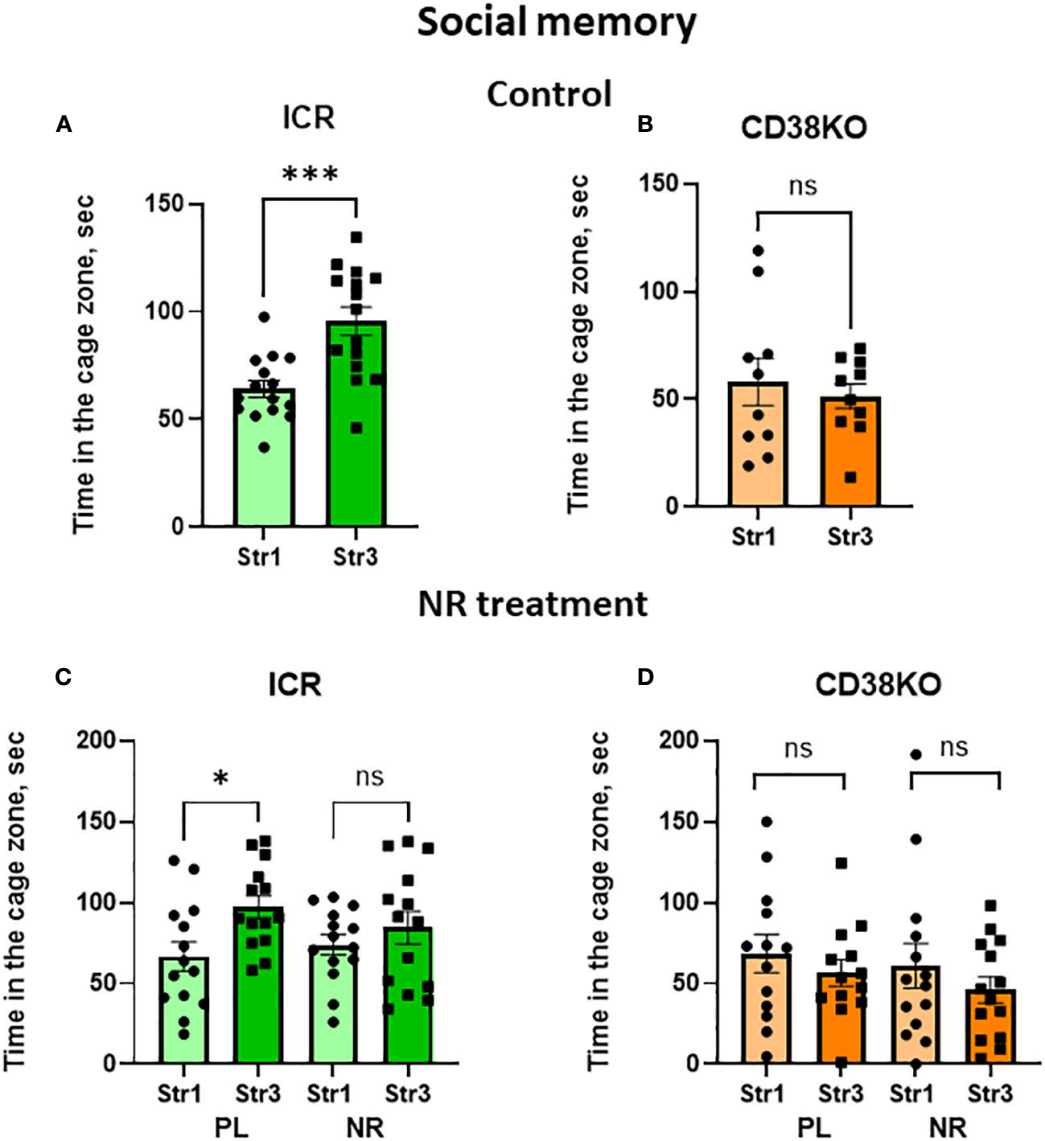
Social memory behavior in wild-type (ICR) and CD38KO mice and no or little effect of nicotinamide riboside (NR) treatment. Duration spent in the chamber with the familiar mouse in a cage (Str1) or a novel mouse (Str3) by ICR **(A, C)** or CD38KO **(B, D)** mice. Mice were treated with gavage administration of saline (PL) or NR for 12 days **(C, D)** or were without any treatment **(A, B)**. Note that social memory behavior was observed in ICR mice **(A, C)** but was not affected by NR treatment **(C)**. Two-tailed Student’s *t*-test, *P < 0.05.

Social short-term memory which was performed with the 30-min separation between trials was observed in wild-type ICR mice (**Figure 7A** **;** two-tailed Student’s *t*-test, *P* < 0.001). CD38KO mice were not able to distinguish between Stranger 1 and Stranger 3 in this test (**Figure 7B**). Interestingly, nicotinamide riboside-treated ICR and CD38KO mice showed a lack of social memory (Two-tailed Student’s *t*-test, *P* > 0.05; **Figures 7C, D**), unlike those treated with saline.

## 4 Discussion

The results showed that CD157KO and CD38KO mice displayed sociability (mouse’s motivation to engage with social (mouse) targets compared to non-social targets), which is an essential characteristic of mice, and gavage administration of nicotinamide riboside had no significant effects on their sociability. We also demonstrated that social preference (social novelty to new social targets) was disrupted in both CD157KO and CD38KO mice, and gavage administration of nicotinamide riboside daily for 12 days ameliorated social preference defects only in CD157KO mice, but not in CD38KO mice. Social memory (preference to novel mice rather than already familiar mice) was displayed in wild-type (ICR strain) mice. This behavior, unfortunately, disappeared after nicotinamide riboside gavage administration. Social memory was not observed in CD38KO (genetic background of the ICR strain) and no social memory was affected by either treatment with saline or nicotinamide riboside. In the current study, the effect of nicotinamide riboside on social memory in CD157KO mice was not investigated, because C57BL6 wild-type mice did not display social memory. Of particular interests, it will be expected to get more sharp results on effects of decreases in NAD concentrations and supplementation effects of nicotinamide riboside at the tissues and behavior levels in double knockout mice which delete both *CD157* and *CD38* genes in future.

Here, apart from confirming the beneficial effects of nicotinamide riboside on social behavior defects in CD157 KO mice, but we also tested its effect on body weight. No apparent effect on body weight was observed during the 12 days of gavage administration. Application of nicotinamide riboside had similar effect as saline in some cases. Such results are important, as they suggest no or little risk by nicotinamide on animal health and fewer side effects.

**Figure 8:**
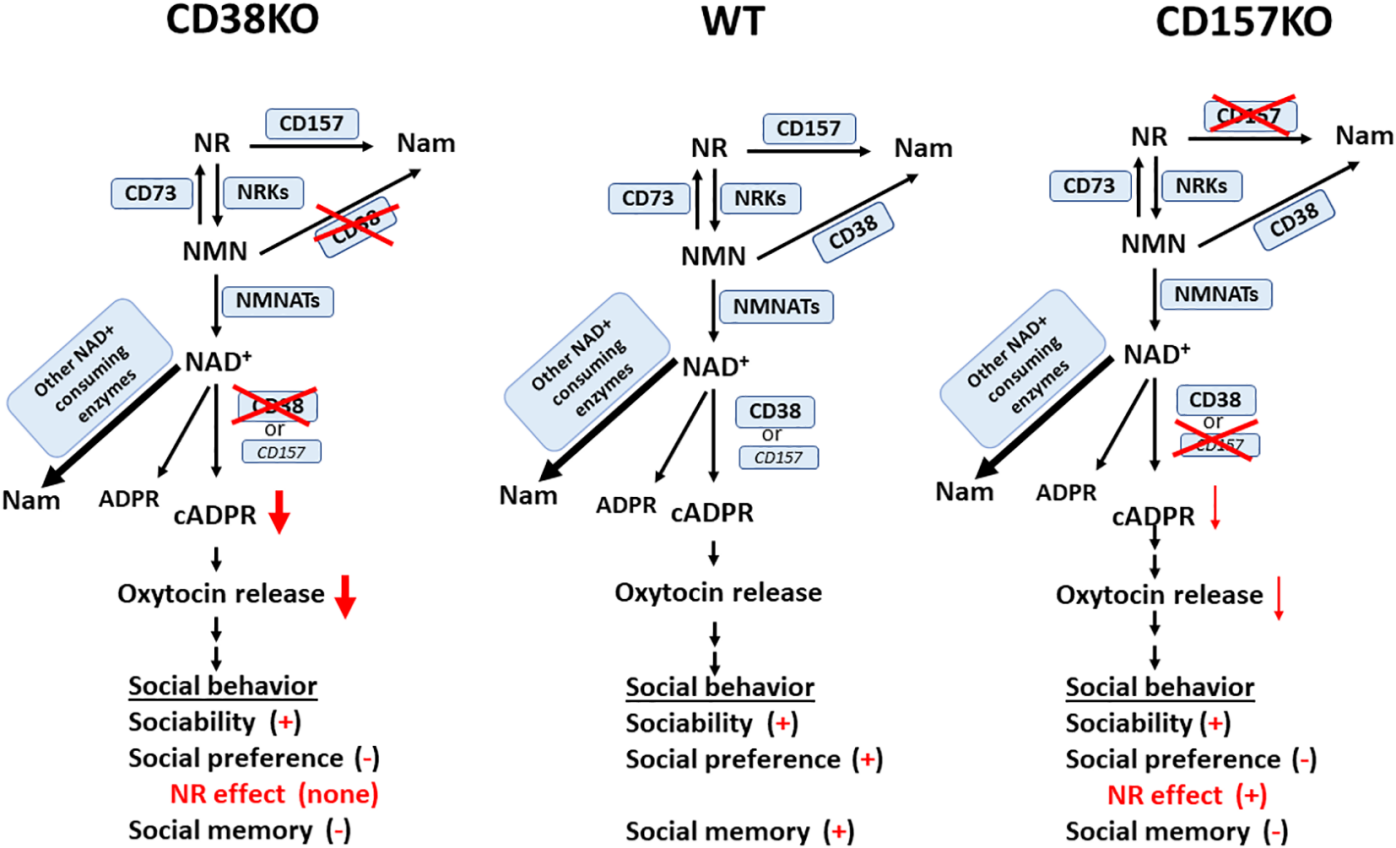
A simplified scheme for possible NAD catabolism from nicotinamide riboside (NR) and metabolism from NAD to cyclic ADP-ribose (cADPR) in wild-type (WT), CD38KO, and CD157KO mice. This is a reciprocal pathway from NR supplementation to NAD and nicotinamide (Nam) *via* nicotinamide mononucleotide (NMN) catalyzed by CD157, CD73 nicotinamide riboside kinases (NRKs), CD38, and nicotinamide mononucleotide adenylyl transferase (NMNATs); NAD is metabolized to Nam, ADP-ribose (ADPR), and cADPR. cADPR production is greatly reduced in CD38KO mice, but less affected in CD157KO mice, which induces different levels of oxytocin release. Social behaviors consist of sociability, social preference, and social memory. Different levels of oxytocin release elicit different levels of impairment of three subclasses of social behaviors. Note that impaired social preference is recovered in CD157KO, but not in CD38KO mice. It is noted that NAD concentration increase in the brain after nicotinamide riboside was confirmed in CD157KO mice (17), but not examined in CD38KO supplemented with nicotinamide riboside.

Nicotinamide riboside is a prospective NAD precursor with high bioavailability (52). Nicotinamide riboside does not demonstrate sirtuin (SIRT) inhibition as a backside effect, unlike nicotinamide (53, 54). Moreover, unlike nicotinamide mononucleotide, nicotinamide riboside can be freely transported across the cell membrane. NAD is synthesized from tryptophan in the *de novo* pathway, as well as from the vitamin precursors, nicotinic acid, nicotinamide mononucleotide, and nicotinamide riboside, in a salvage pathway (38, 39, 55). Thus, it is likely that the exogenous application of nicotinamide riboside nay increase the biosynthesis of NAD through the production of nicotinamide mononucleotide in the first step (**Figure 8**) (40, 41, 56). In terms of elevation of mouse liver NAD, it has been reported that nicotinamide riboside is more efficiently orally bioavailable compared with nicotinamide mononucleotide and nicotinic acid, validating nicotinamide riboside as the favored NAD precursor (52). CD38 catalyzes the pathway of biosynthesis from nicotinamide riboside. Therefore, in agreement to this, the current results are reasonable; CD38 is critical in the recovery of social behavior defects.

NAD concentration increases in the cortex or hypothalamus and oxytocin release to the cerebrospinal fluid by nicotinamide riboside were confirmed in CD157KO mice (17). Although not examined in CD38KO supplemented with nicotinamide riboside, it is highly likely that NAD increase may occur in CD38KO mice as well, because the salvage synthesizing pathway is not disrupted in either KO mice (**Figure 8**). However, in CD38KO mice, the CD38-dependent cADPR system existed in ICR wild-type mice is greatly disrupted, which is why beneficial effects were observed in CD157KO mice but not in CD38KO mice. Existence of CD38 is critical for social behavior, as previously demonstrated (9, 15, 21, 28, 57).

A decrease in volume of the amygdala, an important constituent of the “social brain,” might be caused by a loss of CD157 in the neural stem cells during the developmental stages (14, 21). The behavioral impairments in CD157KO mice were rescued by oxytocin, likely because oxytocin directly targets the intracellular signaling networks in the social brain downstream of CD157, which might be independent of CD38; its downstream signaling networks are indicated in **Figure 8**. Of course, to completely conclude needs to wait until actual measuring of oxytocin release in CD38KO mice treated with nicotinamide riboside. The above observation, however, suggests that oxytocin can be used for the treatment of social avoidance in psychological disorders. Furthermore, whether the results obtained here with mice are applicable to human behavioral recovery is of interest, especially since there have been reports of the impact of nicotinamide riboside on the effectiveness of oxytocin for treating impaired social interaction in cases of ASD. Together, current results suggest nicotinamide riboside is possibly an alternative substitute of oxytocin as a clinical usage (15, 18).

Nicotinamide riboside could be used as a general supplement, potentially for people who have adverse reactions to nicotinic acid or nicotinamide mononucleotide (52). In the brain, the tissue activity of NAD synthesis by nicotinic acid is very low, because nicotinic acid is not suitably recruited with supplement administration (58). Nicotinamide riboside has already been demonstrated as a favorable supplement or therapeutic agent to elevate or maintain cellular NAD contents (39, 42–44). Recently, a study demonstrated an increased consumption of NAD due to increased CD38 in aged subjects and, thus, proposed that inhibition of CD38 or increase in NAD might lead to the longer life span (45, 46). Furthermore, with respect to CD38 inhibition, it would be nice to analyze the area of the big sample of myeloma patients (ranging over thousands) treated with anti-CD38 antibodies: At the moment there are only two approved antibodies, one with total blocking NADase activity (59–61) and a second with partial inhibition of ADP-ribosyl cyclase activity of human CD38 (62, 63).

NAD is an abundant biomolecule and participates in multiple vital processes such as ATP synthesis, redox homeostasis, and signaling pathways (39). These effects can be explained by the enhancement of energy metabolism and activation of SIRT, for which NAD is a co-factor, and by the influence on mTOR pathway regulation (64). NAD as a substrate in ADP-ribosyl cyclase reactions could be a crucial component for oxytocin release induced by nicotinamide riboside. This pathway of elevating of NAD contents may enhance nicotinamide riboside-induced oxytocin release and, thus, influence social behavior. Recent findings suggest that hyper-activation of mTOR may play a crucial role in ASD (65, 66), predicting mTOR as a potential target in ASD therapy. On the other hand, SIRT utilizes NAD as a co-factor and can lead to mTOR pathway inhibition (64). For this, there is evidence that cADP-ribose acts as an endogenous inhibitor of mTOR (67).

In summary, we demonstrated that CD157 and/or CD38 are essential for social behavior. This implies that CD157 and CD38 possess a critical function in the nervous system; they play a role in neuromodulation *via* NAD metabolism, rather than entero-immune regulation (68). Together with benefits in mouse brain tissues induced by supplementation of nicotinamide riboside (17), the current results indicate potential therapeutic applications of nicotinamide riboside in ASD patients, as proposed by Cercillieux et al. (39).

## Data availability statement

The original contributions presented in the study are included in the article/**Supplementary Material**. Further inquiries can be directed to the corresponding author.

## Ethics statement

The animal study was reviewed and approved by the Committee on Animal Experimentation of Kanazawa University Graduate School of Medical Sciences.

## Author contributions

HH and MG designed and performed experiments. HH and MG wrote article and drew figures. All authors contributed to the article and approved the submitted version.
