# Supplementary material for: Remission of social behavior impairment by oral administration of a precursor of NAD in CD157, but not in CD38, knockout mice
Source: Front Immunol. 2023 May 4;14:1166609. doi: 10.3389/fimmu.2023.1166609 (PMC10192747; doi:10.3389/fimmu.2023.1166609)
Supplement: Supplementary file 1 [file Presentation_1.pptx]

## Slide 1
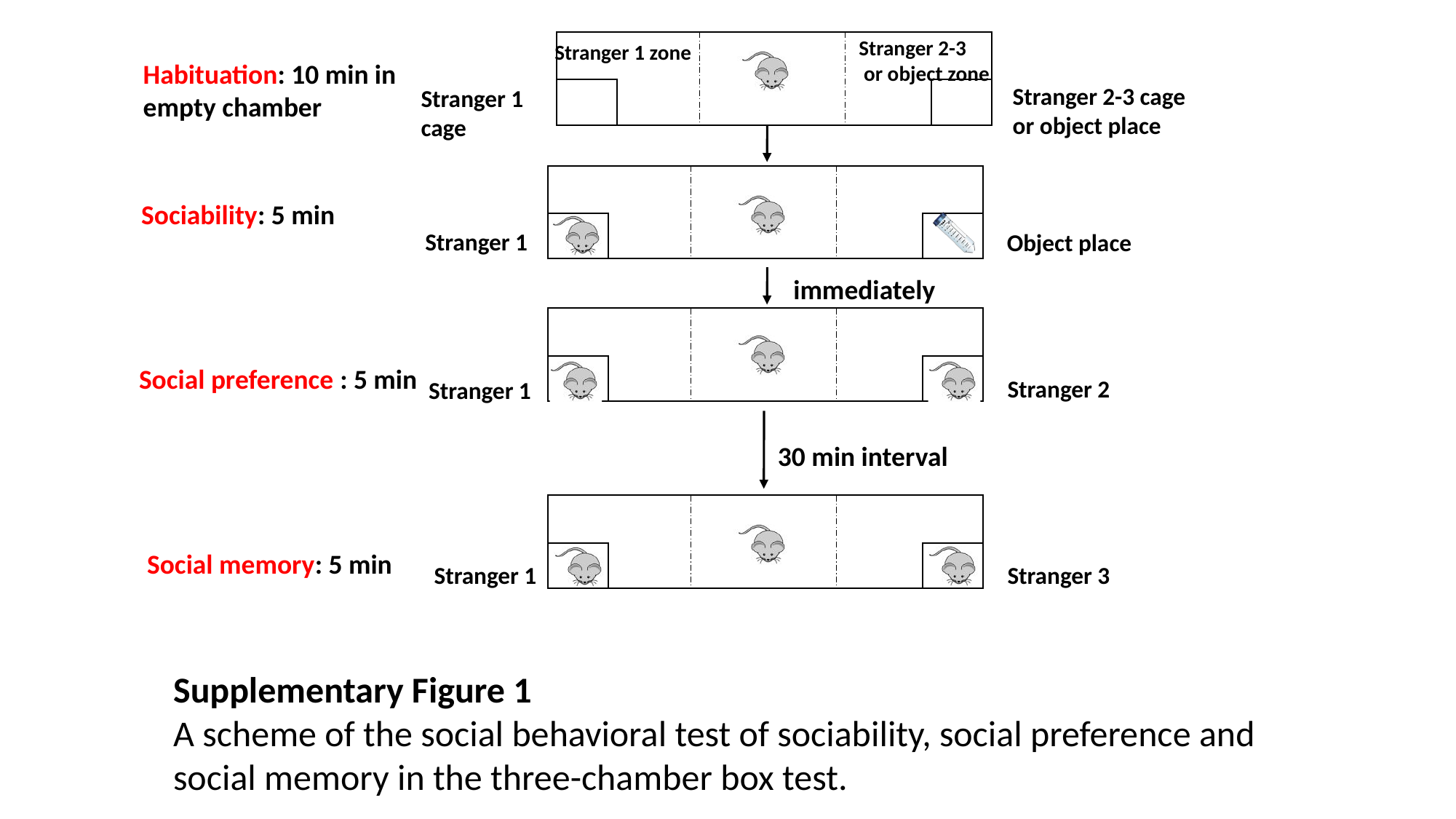

Stranger 2-3
 or object zone
Stranger 1 zone
Habituation: 10 min in
empty chamber
Stranger 2-3 cage
or object place
Stranger 1 cage
Sociability: 5 min
Stranger 1
Object place
immediately
Social preference : 5 min
Stranger 2
Stranger 1
30 min interval
Social memory: 5 min
Stranger 1
Stranger 3
Supplementary Figure 1
A scheme of the social behavioral test of sociability, social preference and social memory in the three-chamber box test.
